# Supplementary material for: De novo cholesterol biosynthesis in bacteria
Source: Nat Commun. 2023 May 22;14:2904. doi: 10.1038/s41467-023-38638-8 (PMC10202945; doi:10.1038/s41467-023-38638-8)
Supplement: Supplementary file 1 — Supplementary Information [file 41467_2023_38638_MOESM1_ESM.pdf]

Supporting Information for:

De novo cholesterol biosynthesis in bacteria

Alysha K. Lee[1], Jeremy H. Wei[1], and Paula V. Welander[1]*

[1]Department of Earth System Science, Stanford University, Stanford, CA 94305, USA

*Corresponding author: welander@stanford.edu

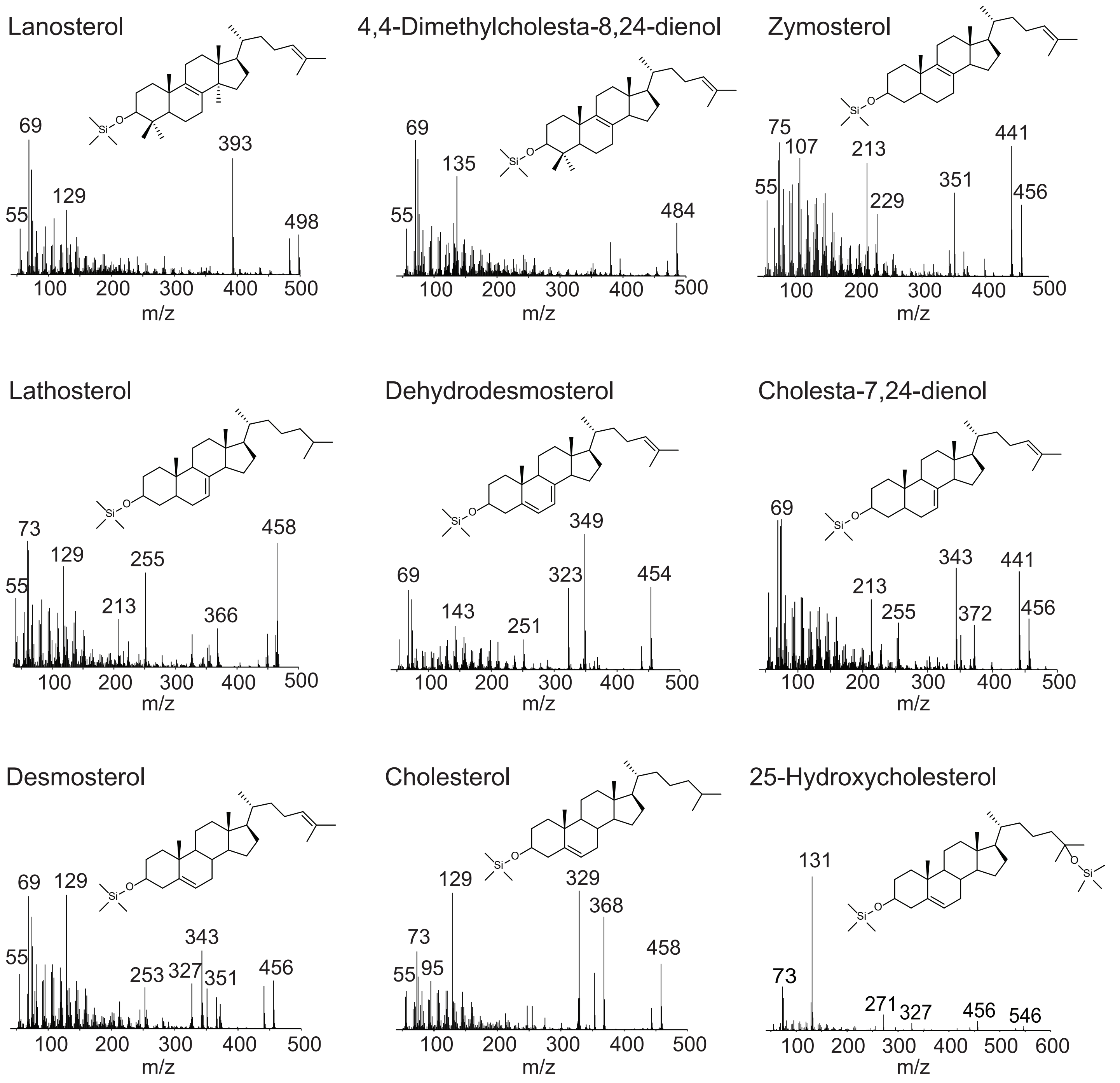

Supplementary Figure 1. Mass spectra of sterols identified in *E. salina* or *Calothrix* NIES-4105 extracts. Extracted sterols were derivatized to trimethylsilyl (TMS) groups and separated on an Agilent 7890B series GC through a 60m Agilent DB17 column (60m x 0.25 mm i.d x 0.1 µm film thickness) with helium as the carrier gas coupled to a 5977A series MS. See Methods for full GC-MS method details.

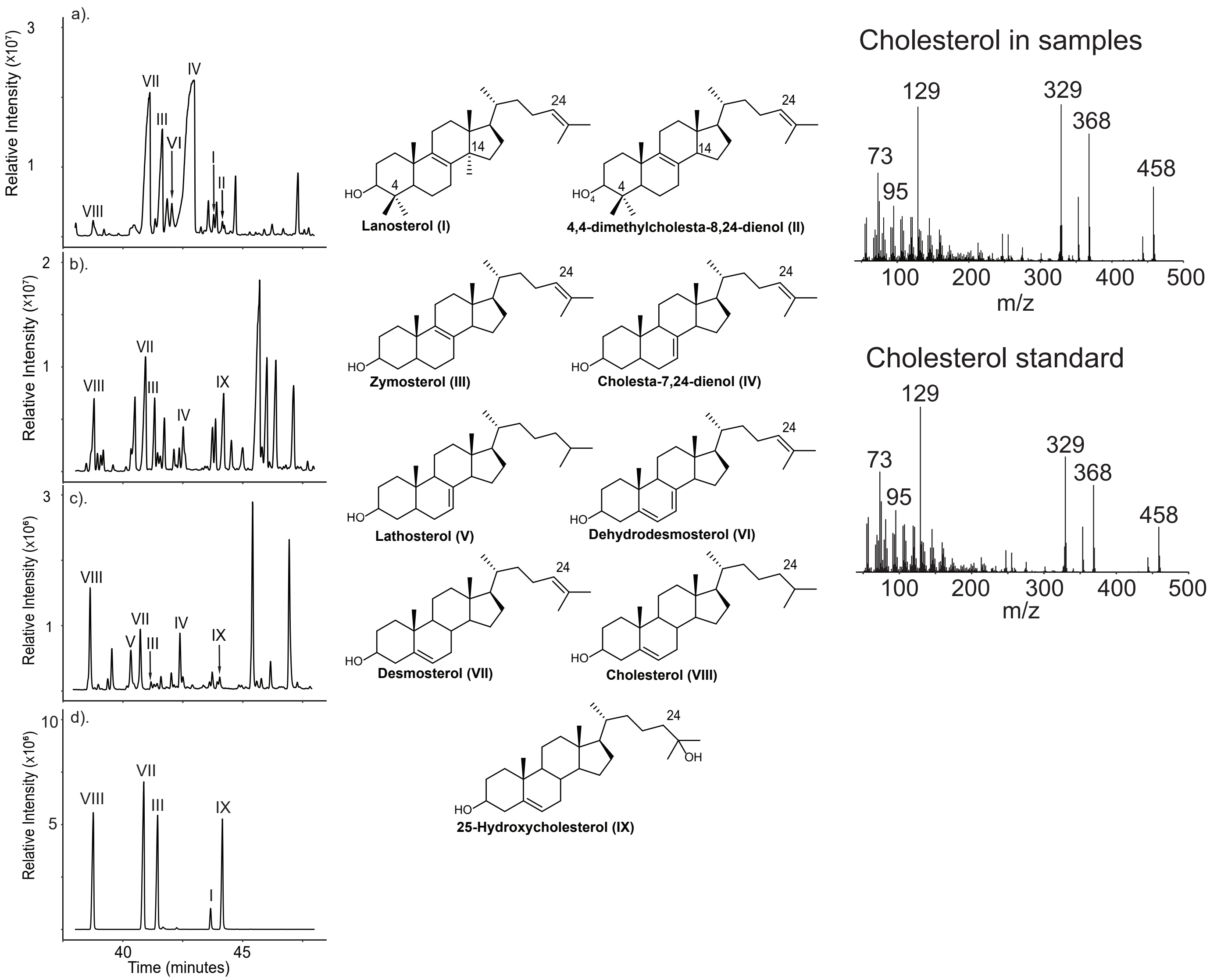

Supplementary Figure 2. Sterol chromats compared to a sterol standard mix. Total ion chromatograms from a). *E. salina* Bligh Dyer and b). acid hydrolysis extractions, c). *Calothrix* acid hydrolysis extraction, and d). a sterol standard mix (lanosterol, zymosterol, desmosterol, cholesterol, and 25-hydroxycholesterol). Mass spectra for the cholesterol standard and the cholesterol found in bacterial extracts are provided for comparison.

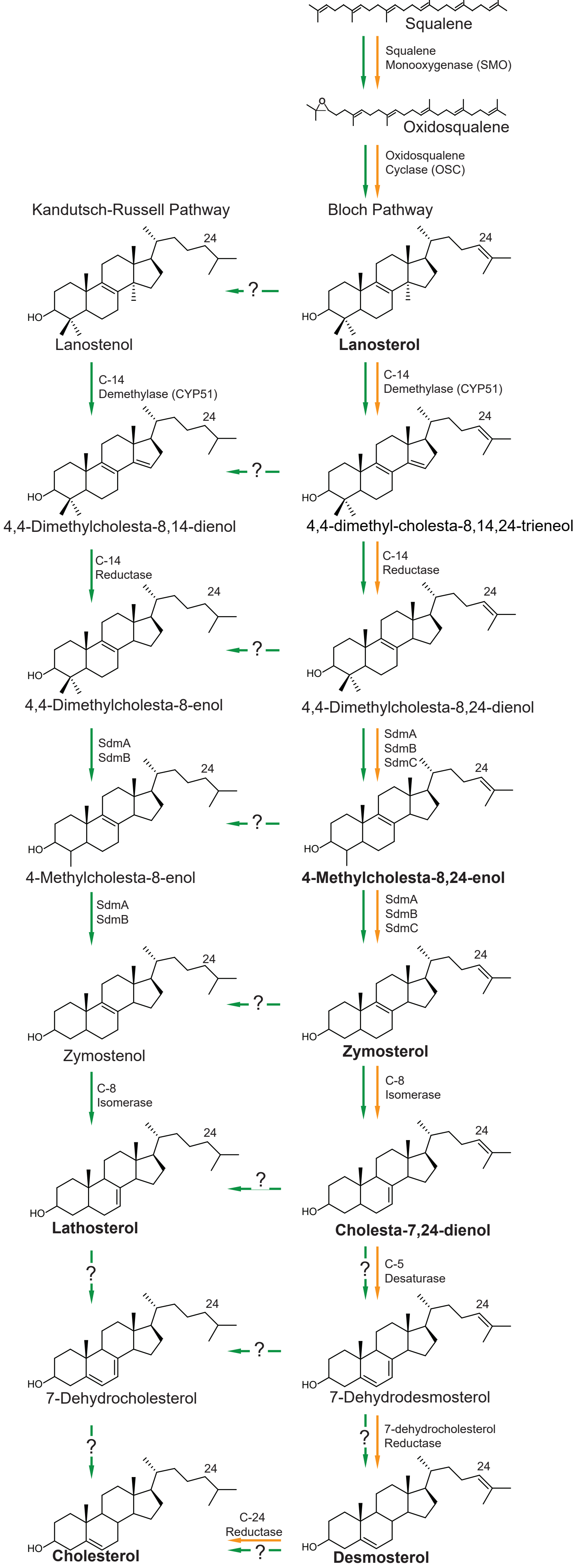

Supplementary Figure 3. Putative cholesterol biosynthesis pathways in *E. salina* (orange arrows) and *Calothrix* (green arrows). The names of intermediates detected in our sterol analysis are bolded. The presence of homologs for each step in cholesterol biosynthesis was determined by BLASTp search (e-value < 1 x e^{-30}). Based on our analysis of cholesterol intermediates, we posit that *E. salina* preferentially uses the Bloch pathway while *Calothrix* uses both the Bloch and Kandutsch-Russell (K-R) pathways. The Bloch pathway proceeds from C-14 and C-4 demethylation through several steps modifying desaturation in the ring structure before saturation at C-24 as the final step. The K-R pathway utilizes the same enzymes to perform cholesterol biosynthesis but C-24 saturation occurs as an intermediary step[1].

Supplementary Table 1. Sterol concentrations for *E. salina* extractions. Quantification methods are described in detail in methods section. Not detected ND. Below quantification limit (BQL).

	**Biomass extracted (mg dry weight)**	**Total lipids extracted(mg)**	**Cholesterol (ng/mg dry weight)**	**Desmosterol (ng/mg dry weight)**	**Zymosterol (ng/mg dry weight)**	**25-OHC (ng/mg dry weight)**
**Bligh Dyer Extraction**						
E.salina culture 1	17.30	1.24	108.38	224.51	36.13	ND
E.salina culture 2	19.43	1.07	70.77	223.70	36.99	ND
E.salina culture 3	16.23	1.43	73.40	348.06	69.13	ND
**Acid Hydrolysis**						
E. salina	73.0	2.8	65.75	87.32	BQL	85.56

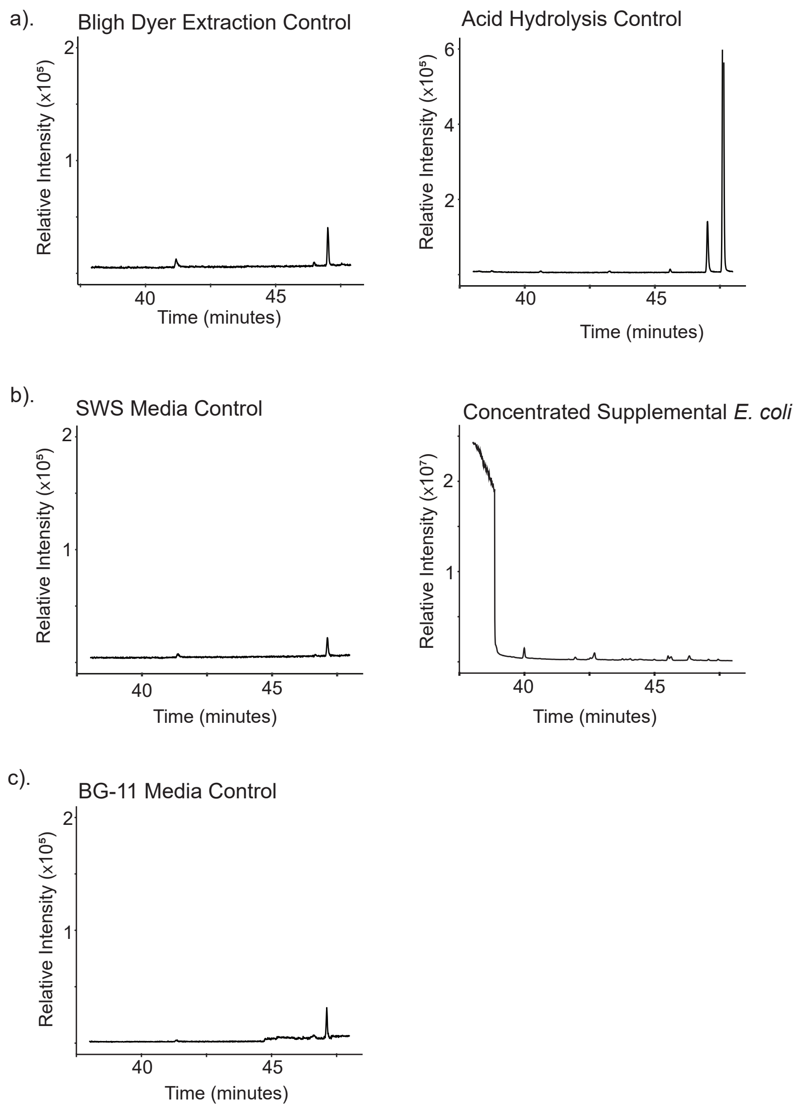

Supplementary Figure 4. Total ion chromatograms of extraction controls and growth media. Total ion chromatogram of total lipid extracts (TLE) from a). extraction processes used to analyze sterols in *E. salina* and *Calothrix*. Sterile water was added in place of bacterial biomass. b). Extractions of media components used to culture *E. salina*. Both medium and supplemental *E. coli* were extracted using a modified Bligh Dyer technique. Extracted *E. coli* was concentrated 5-fold from what was used to culture *E. salina*. c). Extraction of medium used to culture *Calothrix*. Medium was extracted using a modified Bligh Dyer technique. Spectra in peaks of all chromats were compared to the NIST database and published spectra and none were found to be sterols.

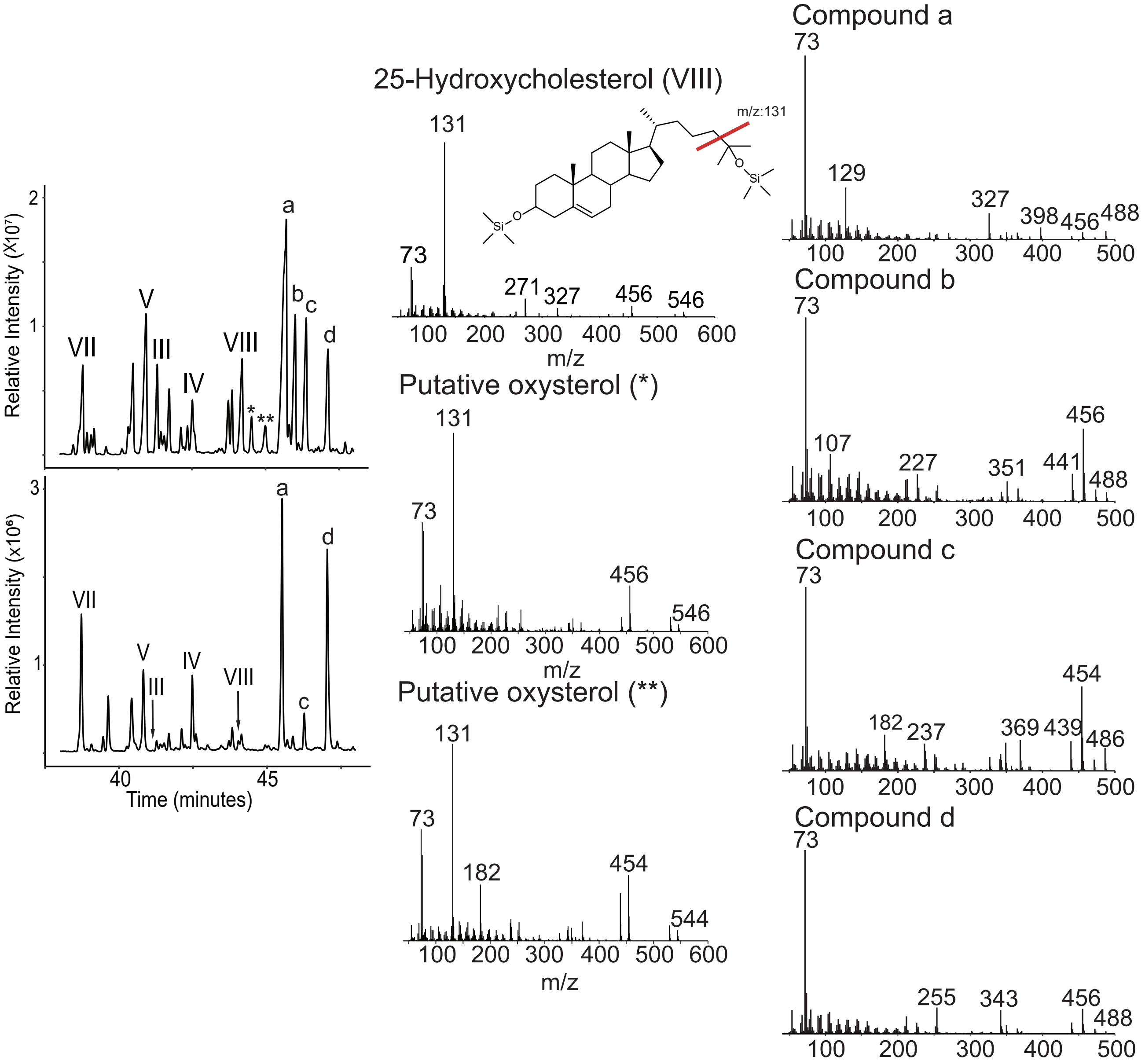

Supplementary Figure 5. Hydroxysterols released from hydrolyzed *E. salina* lipid extracts. TMS-derivatized 25-hydroxycholesterol (25-OCH) has a diagnostic 131 peak that is produced from fragmentation of the side chain. The presence of 25-OCH in *E. salina* samples was confirmed by comparing elution time to a known standard. Two neighboring peaks were found to share the same parent ion and diagnostic 131 peak, suggesting they may also represent sterols hydroxylated at C-25. Additionally, significant relative abundance elute after the 45 minute mark. The prominent 73 peak, also seen in cholesterol diethers, and the parent ion leads us to speculate these may be sterol monoethers, however further characterization is needed to identify these compounds.

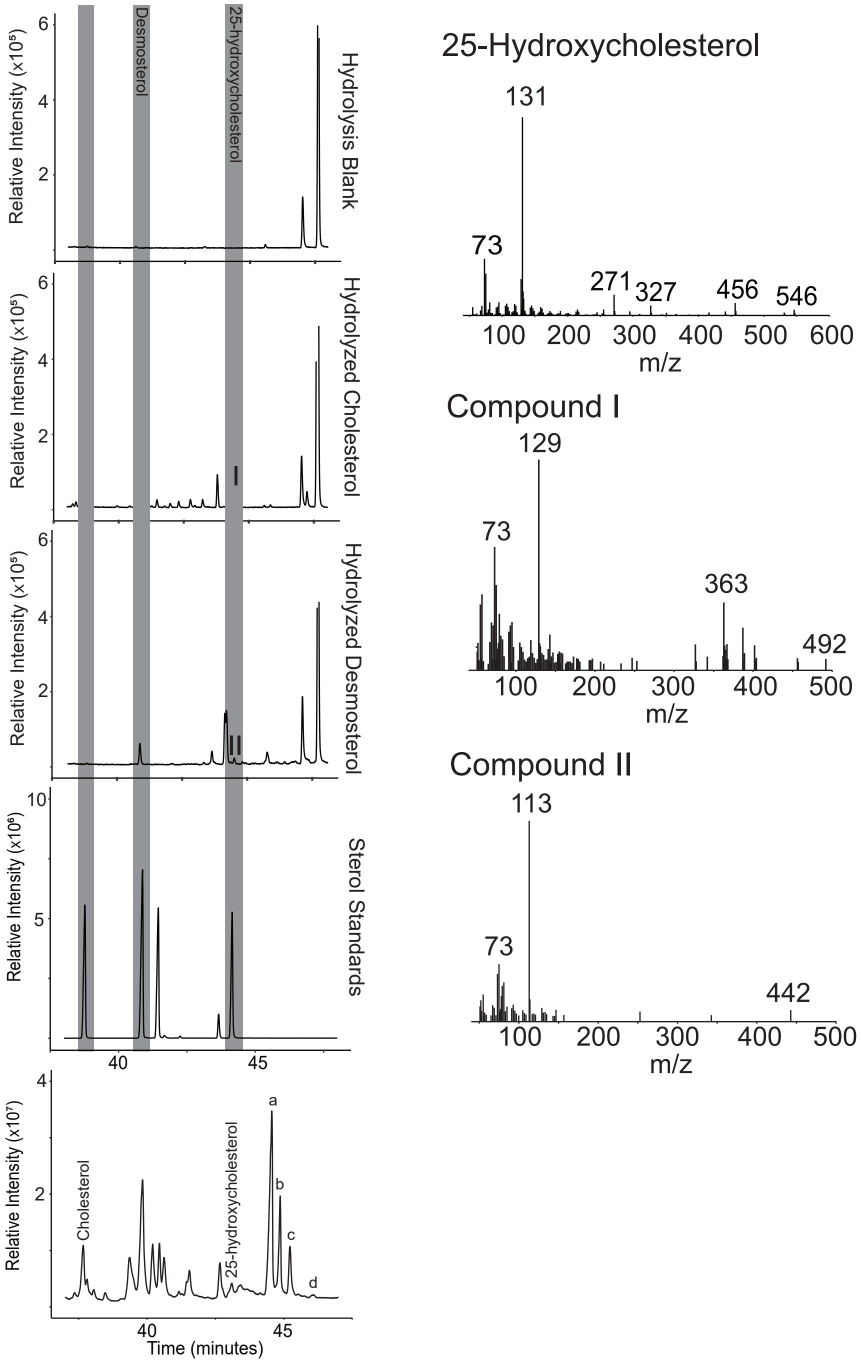

Supplementary Figure 6. Autooxidation controls for acid hydrolysis of sterols. Total ion chromatograms of a hydrolysis blank, a hydrolyzed cholesterol standard, a hydrolyzed desmosterol standard, and *E. salina* biomass hydrolyzed with butylated hydroxytoluene (BHT). Acid hydrolysis was performed as described in the Methods section. Lipids were derivatized to TMS groups. The spectra of peaks observed in the hydrolyzed standard chromats were compared to a 25-hydroxycholesterol standard and spectra deposited in the NIST database and were not found to be oxysterols. Additionally, hydrolysis of *E. salina* biomass in the presence of BHT still resulted in release of 25-hydroxycholesterol and other additional peaks observed in hydrolysis reactions without BHT.

Supplementary Table 2 *E. salina* and *Calothrix* sp. NIES-4105 closest homologs to canonical sterol biosynthesis genes. Sterol biosynthesis genes from *Homo sapiens*, *Saccharomyces cerevisiae*, and *Methylococcus capsulatus* were used as a query for BLASTp search (e-value < 1 x $e^{0.0}$) to identify putative cholesterol biosynthesis pathways in *E. salina* and *Calothrix*. Putative sterol biosynthesis genes in each organism were then used as a query for BLASTp search in the other bacterium. IMG database locus tags for top hits in each bacterium, with corresponding e-values and identities, are listed for each gene in the canonical cholesterol biosynthesis pathway. E-values colored green and bolded within our e-value cutoff (e-value < 1 x e^{-30}). E-values colored yellow and marked with an asterisk denote homologs where lowest e-value hit in either *Calothrix* or *E. salina* was not the putative sterol biosynthesis gene identified through previous BLASTp searches. N/A denotes organisms without listed sterol biosynthesis gene. --- denotes no homolog found with set e-value cutoffs.

	H. sapiens	***S. cerevisiae***	***M. capsulatus***	***E. salina***	***Calothrix* sp. NIES-4105**
E. salina					
Squalene monooxygenase (Ga0097779_102654)	$1e^{-27}$/25%	$2e^{-28}$/24%	**$2e^{-37}$/29%**	N/A	**$3e^{-40}$/31%**
Oxidosqualene cyclase (Ga0097779_114516)	**$6e^{-112}$/33%**	**$3e^{-96}$/30%**	**$1e^{-169}$/44%**	N/A	**$8e^{-155}$/38%**
C-14 demethylase (Ga0097779_103211)	**$4e^{-97}$/36%**	**$7e^{-86}$/34%**	**$8e^{-133}$/44%**	N/A	**$1e^{-154}$/48%**
C-14 reductase (Ga0097779_105926)	**$1e^{-42}$/32%**	**$2e^{-41}$/29%**	N/A	N/A	**$5e^{-44}$/63%**
SdmA (Ga0097779_103152)	N/A	N/A	**$3e^{-104}$/45%**	N/A	**$1e^{-163}$/60%**
SdmB (Ga0097779_103151)	N/A	N/A	**$2e^{-79}$/41%**	N/A	**$1e^{-121}$/50%**
ERG25 (Ga0097779_103191)	$2e^{-08}$/24%	$2e^{-15}$/26%	N/A	N/A	$5e^{-27}$/30%
ERG26 (Ga0097779_105318)	**$2e^{-46}$/32%**	**$1e^{-39}$/32%**	N/A	N/A	**$3e^{-44}$/31%**
ERG27 (Ga0097779_11785)	$8e^{-12}$/26%	---	N/A	N/A	$4e^{-11}$/27%*
C-8 sterol isomerase (Ga0097779_103215)	**$2e^{-37}$/39%**	**$8e^{-47}$/43%**	N/A	N/A	**$2e^{-57}$/51%**

C-5 desaturase (Ga0097779_11078)	**1e^{-45}/35%**	**1e^{-41}/36%**	N/A	N/A	4e^{-16}/30%*
7-dehydrocholesterol reductase (Ga0097779_100662)	**1e^{-87}/36%**	---	N/A	N/A	2e^{-16}/30%*
Delta 24 sterol reductase (Ga0097779_101115)	**2e^{-127}/41%**	---	**2e^{-94}/38%**	N/A	---
***Calothrix* sp. NIES-4105**					
Squalene monooxygenase (Ga0263810_115391)	3e^{-27}/25%	1e^{-28}/27%	**3e^{-32}/26%**	**2e^{-40}/31%**	N/A
Oxidosqualene cyclase (Ga0263810_115390)	**2e^{-163}/39%**	**3e^{-105}/32%**	**0.0/44%**	**9e^{-155}/38%**	N/A
C-14 demethylase (Ga0263810_115381)	**4e^{-111}/38%**	**1e^{-78}/33%**	**e^{-131}/44%**	**2e^{-158}/48%**	N/A
C-14 reductase (Ga0263810_115392)	2e-^{22}/40%	2e^{-15}/36%	N/A	**2e^{-42}/63%**	N/A
SdmA (Ga0263810_115380)	N/A	N/A	**1e^{-102}/44%**	**1e^{-163}/60%**	N/A
SdmB (Ga0263810_115382)	N/A	N/A	**7e^{-97}/45%**	**1e^{-121}/50%**	N/A
ERG25 (Ga0263810_118348)	3e^{-15}/32%	3e^{-18}/35%	N/A	1e^{-28}/30%*	N/A
ERG26 (Ga0263810_118461)	**7e^{-41}/31%**	**8e^{-33}/30%**	N/A	3e^{-44}/31%*	N/A
ERG27 (Ga0263810_119321)	2e^{-16}/25%		N/A	2e^{-13}/27%	N/A
C-8 sterol isomerase (Ga0263810_115389)	**3e^{-31}/35%**	5e^{-27}/36%	N/A	**2e^{-57}/49%**	N/A
C-5 desaturase (Ga0263810_118508)	3e^{-23}/36%	4e^{-08}/25%	N/A	1e^{-21}/29%	N/A
7-dehydrocholesterol reductase (Ga0263810_115392)	2e^{-16}/31%	---	N/A	6e^{-14}/30%	N/A
Delta 24 sterol reductase (Ga0263810_116277)	2e^{-10}/34%	----	8e^{-11}/31%	6e^{-04}/25%	N/A

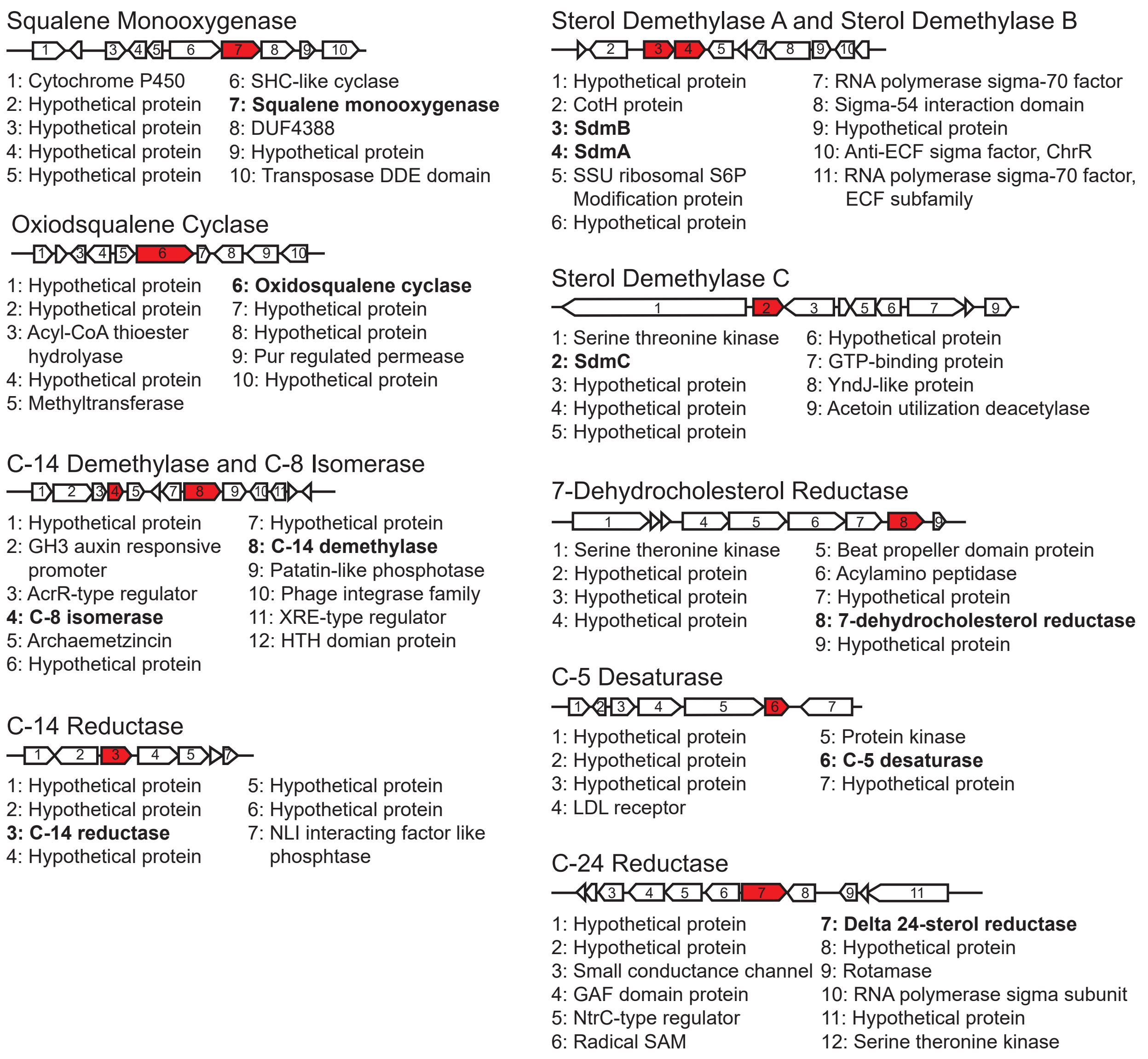

Supplementary Figure 7. Genomic neighborhoods of sterol biosynthesis genes in *E. salina*. Sterol biosynthesis genes identified by our BLASTp search (<1xe $^{-30}$; 30% ID) are colored red and text labels bolded.

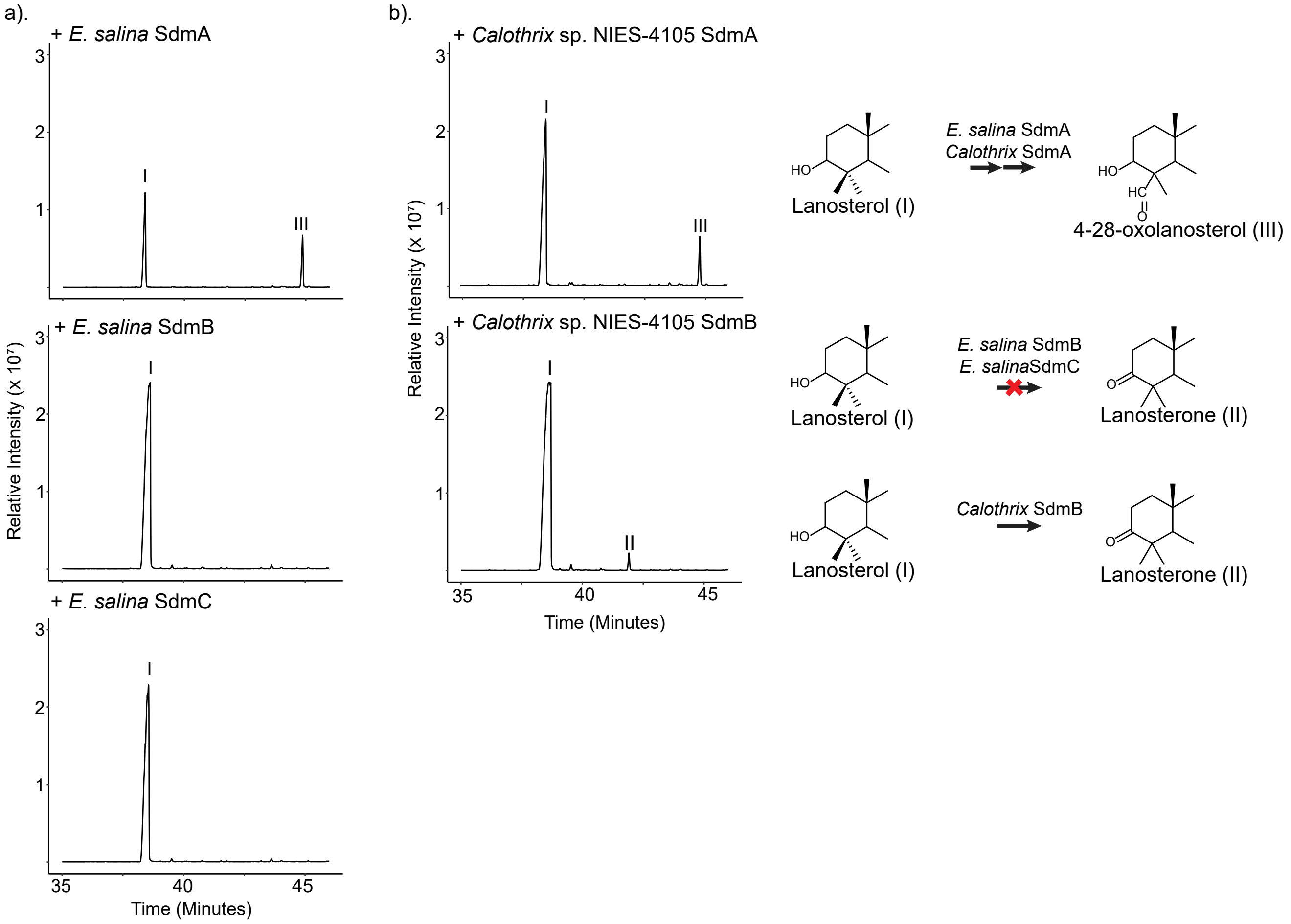

Supplementary Figure 8. Total ion chromatograms of TLEs of a). heterologous expression strains from *E. salina* SdmA, SdmB, and SdmC homologs and b). heterologous expression strains from *Calothrix* SdmA and SdmB homologs. In both organisms, SdmA homologs produce the C-4 oxidation intermediate, 4-28-oxolanosterol. The SdmB and SdmC homologs from *E. salina* have no apparent effect on the substrate lanosterol. The SdmB homolog from *Calothrix* oxidizes the 3β-hydroxyl into a ketone, producing lanosterone.

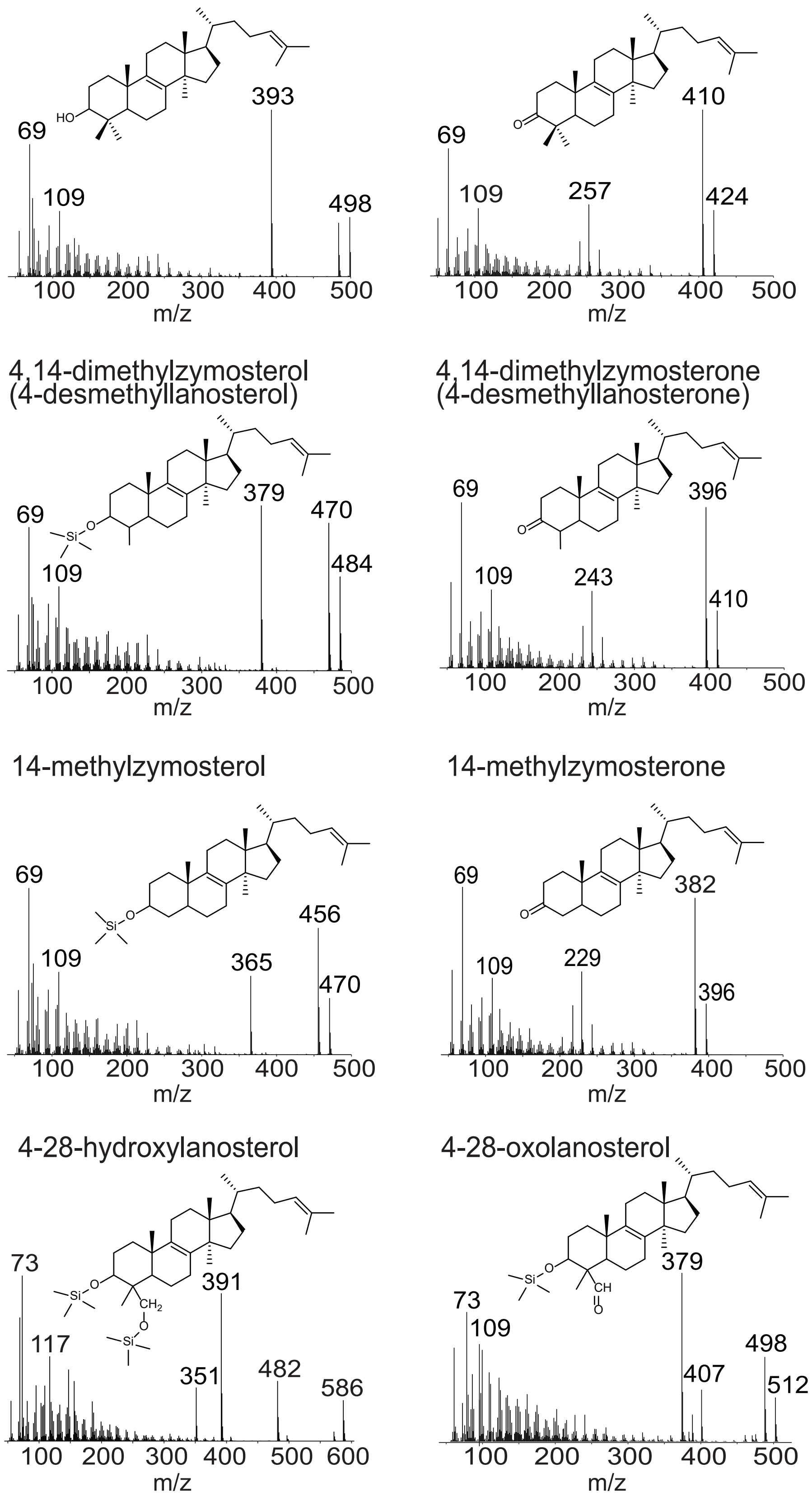

Supplementary Figure 9. Mass spectra of sterols identified in heterologous expression cultures. Extracted sterols were derivatized to TMS groups and separated on an Agilent 7890B series GC with helium as the carrier gas and was coupled to a 5977A series MS. See Methods section for full GC-MS method details.

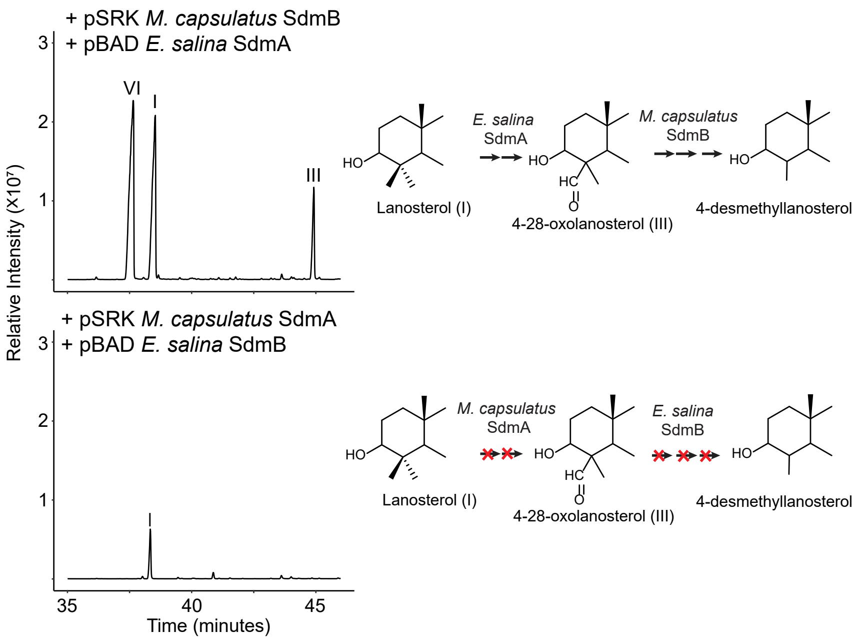

Supplementary Figure 10. Heterologous expression of reciprocal SdmA and SdmB pairs from *E. salina* and *M. capsulatus*. SdmA and SdmB homologs from either *E. salina* or *M. capsulatus* were expressed from compatible plasmids in an *E. coli* strain engineered to overproduce lanosterol. Expression of *E. salina* SdmA with *M. capsulatus* SdmB resulted in single demethylation at C-4 while expression of *E. salina* SdmB with *M. capsulatus* SdmA did not, suggesting the *E. salina* SdmB homolog is insufficient to carry out the decarboxylation and reduction steps required to demethylate at C-4.

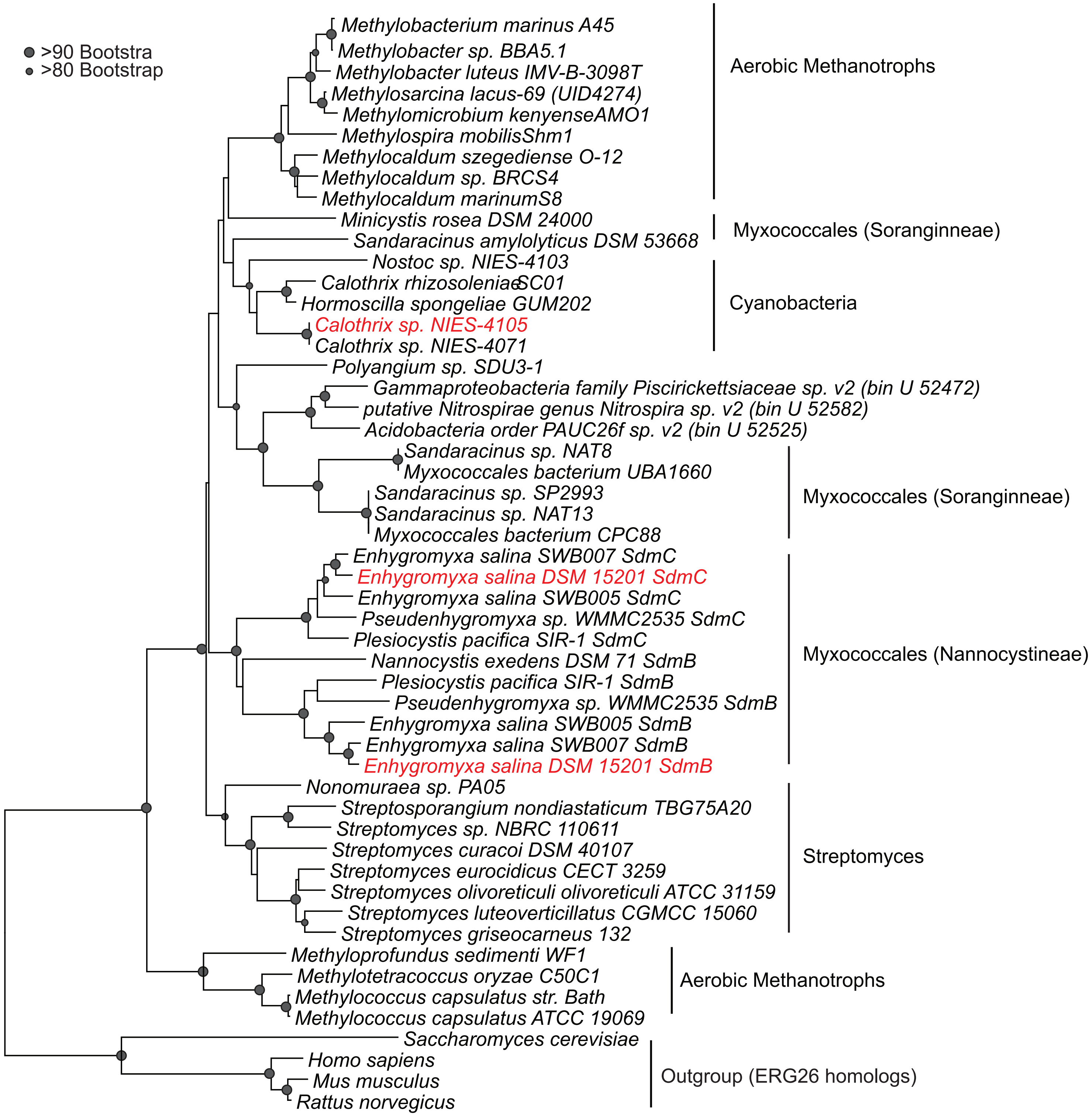

Supplementary Figure 11. Neighbor-joining phylogenetic tree of SdmBC homologs. Homologs were identified by BLASTp search (e-value <1 x e^{-50}; 30% identity) of the JGI genomic databases. Protein sequences were aligned by MUSCLE using MEGA. A neighbor-joining tree was generated using the gamma model, four gamma rate categories and 500 bootstrap replicates. SdmB homologs from *E. salina* and *Calothrix* and the SdmC homolog from *E. salina* tested in this paper are highlighted red. SdmC homologs are only present in the Myxococcata suborder Nannocystineae and form a monophyletic clade with SdmB homologs from the same suborder.

a). C-4 demethylation in eukaryotes (fungi and vertebrates):

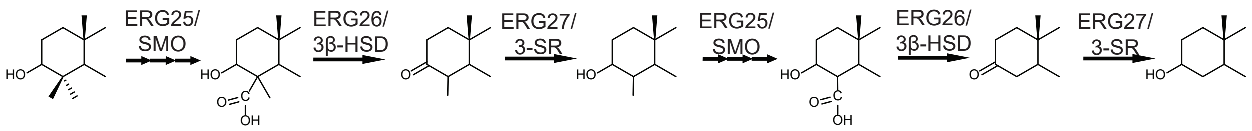

b). C-4 demethylation in aerobic methanotrophic bacteria:

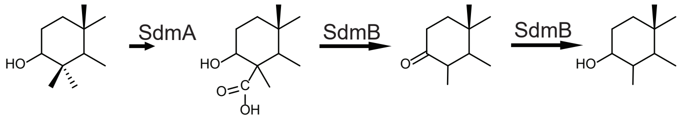

c). C-4 demethylation in *E. salina*:

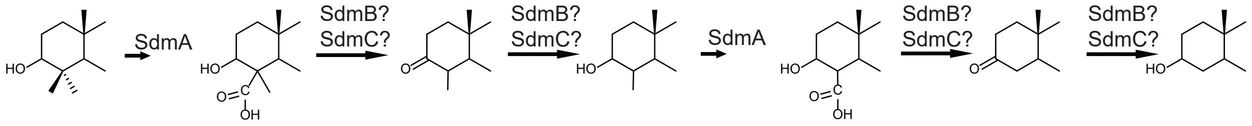

d). C-4 demethylation in *Calothrix*:

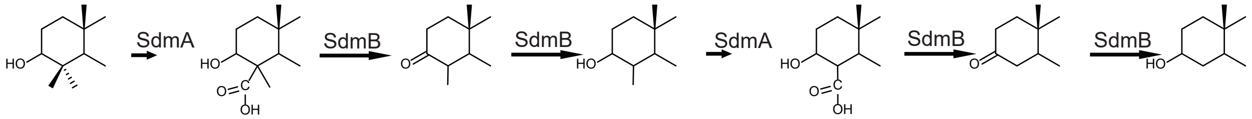

Supplementary Figure 12. C-4 demethylation in eukaryotes and bacteria. a). In eukaryotes, C4 demethylation is carried out by three enzymes. ERG25/SMO, a monooxygenase, performs three oxidation reactions to carboxylate the 4α-methyl group. ERG26/3β-HSD, a short chain dehydrogenase reductase (SDR)-type reductase, catalyzes the decarboxylation of the 4amethyl group and remaining methyl group epimerizes into the α-position. ERG27/3-SR, an SDRtype reductase, reduces the C-3 ketone to a hydroxyl. This concerted reaction repeats a second time to remove the remaining methyl group. b). In aerobic methanotrophs, SdmA, a dioxygenase, performs the required oxidation reactions to carboxylate the 4β-methyl group.

SdmB, an SDR-type reductase, then both decarboxylates the 4β-methyl group and reduces the C-3 ketone to a hydroxyl, producing the final monomethyl product. c). In *E. salina*, SdmA oxidizes one of the C-4 methyl groups to a carboxylate. SdmB and/or SdmC then perform the decarboxylation and reduction reactions. Further mechanistic study is required to untangle the role these two proteins have in C-4 demethylation. This reaction then repeats likely through the same mechanism. d). In *Calothrix*, SdmA oxidizes one of the methyl groups to a carboxylate. SdmB then performs the decarboxylation and reduction reactions to remove the first methyl group. This reaction then repeats, likely through the same mechanism.

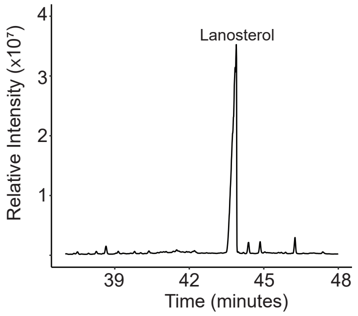

Supplementary Figure 13. Total ion chromatogram of TLE from heterologous expression of oxidosqualene cyclase (*osc*) from serial passaged *Calothrix* strain. *osc* was amplified from genomic DNA extracted from the serial passaged *Calothrix* strain, cloned into a compatible plasmid and expressed in an *E. coli* strain engineered to overproduce oxidosqualene. This resulted in production of lanosterol, demonstrating that when expressed *osc* from the serial passaged *Calothrix* strain is functional.

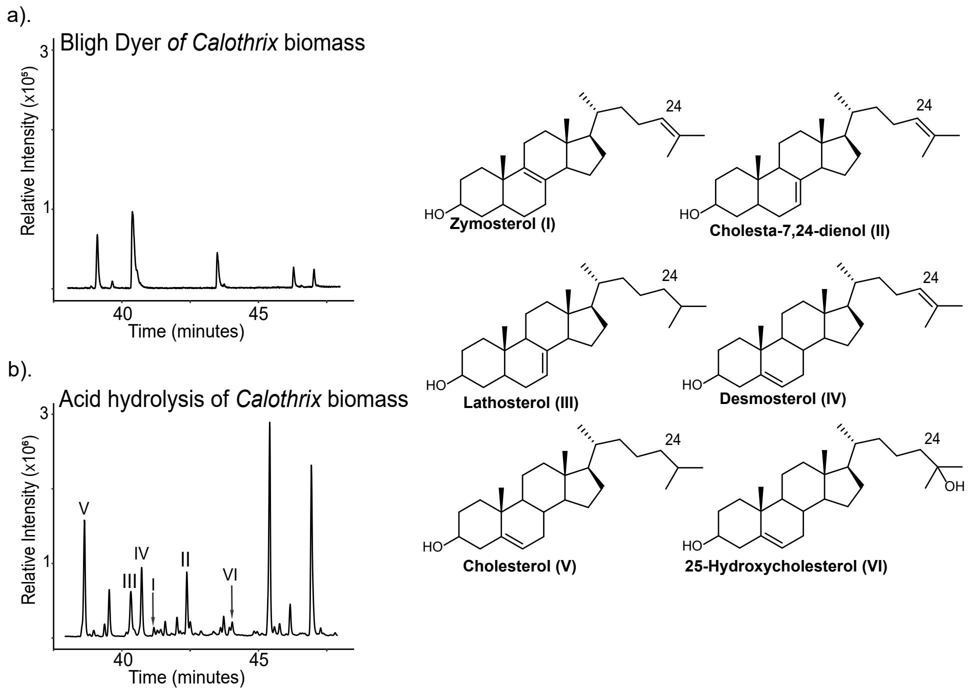

Supplementary Figure 14. Initial sterol analysis of *Calothrix* sp. NIES-4105. a). Total ion chromatograms of free lipids extracted from *Calothrix* using a modified Bligh Dyer procedure. Peaks present in this chromatogram were not sterols based on retention time and comparison to a sterol standard mix (lanosterol, zymosterol, desmosterol, cholesterol, and 25-hydroxycholesterol at 40 ng/μl). b). Total ion chromatogram of ether and ester bound lipids extracted from *Calothrix* biomass. Sterol identified included cholesterol (V) and both C-24 saturated (III) and unsaturated intermediates (I, II, and IV). Additionally, hydrolysis released 25-hydroxycholesterol (VI). All lipids were derivatized to TMS groups. Mass spectra of identified sterols are shown in Supplementary Figure 1.

Supplementary Table 3. Mutations present in serial passaged *Calothrix* sp. NIES-4105. RA indicates read alignment evidence for mutation. JC indicates new junction evidence for mutation. Mutation column describes specific mutation. In intergenic mutations, the distance from the start (+) or stop (-) of nearest genes are listed. In coding mutations, the location of mutation in gene as well as original nucleotide length is listed. IMG locus tags and annotation for each gene or nearest genes are listed.

Evidence	Mutation	Annotation	Gene	Description
RA	Δ1 bp	intergenic (+33/-65)	*118839* → / → *118838*	hypothetical protein/hypothetical protein
RA	$(C)_{11\rightarrow10}$	intergenic (-194/+40)	*114372*← / ← *114371*	hypothetical protein/hypothetical protein
RA	C→G	intergenic (-346/+392)	*114310*← / ← *114309*	3-octaprenyl-4-hydroxybenzoate carboxy-lyase/3'-5' exoribonuclease, VacB and RNase II
RA	2 bp→GA	intergenic (-385/+352)	*114310*← / ← *114309*	3-octaprenyl-4-hydroxybenzoate carboxy-lyase/3'-5' exoribonuclease, VacB and RNase II
RA	+G	coding (2515/2589 nt)	*114218* ←	TPR repeat-containing protein
RA	4 bp→AGAA	coding (2511-2514/2589 nt)	*114218*←	TPR repeat-containing protein
RA	G→C	L709L (CTC→CTG)	*113884* ←	TPR repeat-containing protein
JC	+34 bp	coding (2106/2169 nt)	*113884* ←	TPR repeat-containing protein
RA	Δ1 bp	coding (991/1116 nt)	*113884* ←	hypothetical protein
JC	13 bp→130 bp	coding (579591/951 nt)	*113087*←	hypothetical protein
RA	T→G	intergenic (+303/+143)	*112194* → / ← *112193*	hypothetical protein/hypothetical protein
RA	T→C	intergenic (+323/-312)	*111735* → / → *11734*	50S ribosomal protein L25/hypothetical protein

RA	T→G	intergenic (+328/-307)	*111735* → / → *11734*	50S ribosomal protein L25/hypothetical protein
RA	A→T	intergenic (-445/-271)	*119725* ← / → *119724*	hypothetical protein/hypothetical protein

Supplemental Table 4. Bacterial strains used in this study.

**Strain**	**Genotype/Description**	**Source**
Enhygromyxa salina DSM 15201	Wild type DSM 15201	DSMZ
Calothrix sp. NIES-4105	Wild type NIES-4105	NIES
Escherichia coli DH10B	Strain used for constructing plasmids, heterologous expression, and culturing *E. salina.*<br><br>F^-endA1 recA1 galE15 galK16 nupG rpsL ΔlacX74 Φ80lacZΔM15 araD139 Δ(ara,leu)7697 mcrA Δ(mrr-hsdRMSmcrBC)$λ^-$	Invitrogen

Supplemental Table 5. Oligonucleotides used in this study. F indicates forward primer, R indicates reverse primer, seq indicates sequencing primer, MCAT indicates *Methylococcus capsulatus*, ESA indicates *Enhygromyxa salina*, and CALO indicates *Calothrix* sp. NIES_4105.

**Oligonucleotide**	**Sequence**	**Notes**
AL1	CAATTTCACACAGGAGGCAAGCATATGAGC CGATCGATCAGAAAC	SLIC-pSRK-NdeI-MCAT *sdmA* F
AL2	CGCGCTTGGCGTAATCATGGTCATCATGCC GGGTCTGCC	SLIC-pSRK-NdeI-MCAT *sdmA* R
AL3	CAATTTCACACAGGAGGCAAGCATATGACC ACACTGGTCACCGGC	SLIC-pSRK-NdeI-MCAT *sdmB* F
AL4	GCGCTTGGCGTAATCATGGTCATCAGATCA TCCCCCTCTCCCT	SLIC-pSRK-NdeI-MCAT *sdmB* R
AL5	AATGCAGCTGGCACGACAGG	pSRK seq F
AL7	CCAGGGTTTTCCCAGTCAC	pSRK seq R
AL22	CCGCCAGGCAAATTCTGTTT	pBAD seq R
AL23	CGTCACACTTTGCTATGCCA	pBAD seq F
AL34	TTCTTTCCGAAGGCGTCGC	ESA *sdmB* seq
AL35	TTGCCCCAAAAGGTGACCTCG	ESA *sdmA* seq
AL99	TTGGGCTAGCAGGAGGAATTCACATGTCTA CCAAAGTTCGCATCCCC	SLIC-pBAD-NcoI-ESA *sdmA* F
AL100	GACTCTAGAGGATCCCCGGGTAC TCACGACCCGCTGGGC	SLIC-pBAD-NcoI-ESA *sdmA* R
AL95	TTGGGCTAGCAGGAGGAATTCACATGAGTG AAGCCGAACCCACAG	SLIC-pBAD-NcoI-ESA *sdmB* F
AL96	GACTCTAGAGGATCCCCGGGTACTTACTCG GCGGCTTCGGT	SLIC-pBAD-NcoI-ESA *sdmB* R
AL111	CTGTGGGTTCGGCTTCACTCATTTACGACC CGCTGGGCA	SLIC-pBAD-NcoI-ESA *sdmA*-overlap w *sdmB*-R
AL112	TGCCCAGCGGGTCGTAAATGAGTGAAGCCG AACCCACAG	SLIC-pBAD-NcoI-ESA *sdmB*-overlap w *sdmA*-F
AL142	GCCCGGGGGATCCACTAGTTTCAGTGTCGG CTCGAGAT	SLIC-pBAD-XbaI-ESA *sdmC* R
AL143	CCACCGCGGTGGCGGCCGCTATGGCTGAC CCAGCGTAT	SLIC-pBAD-XbaI-ESA *sdmC* F
AL161	TTCGCGCGCTGTTTCAGCC	ESA sdmC seq
AL198	TTGGGCTAGCAGGAGGAATTCACATGAAAG ACATAGCTATAAAAGGCG	SLIC-pBAD-NcoI-CALO *sdmA* F

AL199	GACTCTAGAGGATCCCCGGGTACCTAGTTT GGGGCGCTCG	SLIC-pBAD-NcoI-CALO *sdmB* R
AL200	CTAAGTTTTCTGACTGTTGA	CALO sdmAB Seq
AL258	GACTCTAGAGGATCCCCGGGTACTCAACAG TCAGAAAACTTAGCCTCA	SLIC-pBAD-NcoI-CALO *sdmA* R
AL259	AGCTCCCGTTACCAGAATTGTCATTCAACAG TCAGAAAACTTAGCCTCA	Overlap PCR CALO *sdmA* R
AL260	TTGGGCTAGCAGGAGGAATTCACATGACAA TTCTGGTAACGGGAG	SLIC-pBAD-NcoI-CALO *sdmB* F
AL261	GCTAAGTTTTCTGACTGTTGAATGACAATTC TGGTAACGGGAG	Overlap PCR CALO *sdmB* F
AL264	CAATTTCACACAGGAGGCAAGCATATGTCT GAACATTTAAACACCAAAC	SLIC-pBAD-NcoI-CALO *osc*-F
AL265	CGCGCTTGGCGTAATCATGGTCATCATCAA ACCATTCGTTTCAACCG	SLIC-pBAD-NcoI-CALO *osc*-R

Supplementary Table 6. Plasmids used in this study. (*) indicates this study, RBS indicates ribosome binding site, MCAT indicates *Methylococcus capsulatus*, ESA indicates *Enhygromyxa salina*, and CALO indicates *Calothrix* sp. NIES_4105.

Plasmid	Description	Reference
pTrc-*sqs*-synRBS-*osc*-synRBS-*smo* (pABB501)	MEALZ_3096-MEALZ_0768-MEALZ_0767 (Squalene synthase-Oxidosqualene cyclase-Squalene epoxidase) optimized expression plasmid (altered osc and smo RBSs).	Lee et al, 2018[2]
pSRKGm-lacUV5-rbs5 (pABB492)	pBBR1 ori, lacUV5 promoter, Gmr	Banta et al, 2017[3]
pBAD1031K (pABB466)	pRV1031 ori, pBAD promoter, Kanr,	Chakravartty and Cronan, 2015[4]
pSRK_MCAT_*sdmA* (pAL7004)	H156DRAFT_2756 (*sdmA*) expression plasmid<br><br>H156DRAFT_2756 was amplified by PCR with primers AL1 and AL2. The fragment was assembled by SLIC into the NdeI site of pABB492. Sequence was confirmed with oligos AL5 and AL7.	(*)
pSRK_MCAT_*sdmB* (pAL7003)	H156DRAFT_2755 (*sdmB*) expression plasmid<br><br>H156DRAFT_2755 was amplified by PCR with primers AL3 and AL4. The fragment was assembled by SLIC into the NdeI site of pABB492. Sequence was confirmed with oligos AL5 and AL7.	(*)
pBAD1031K_ESA_*sdmA* (pAL7134)	Ga0097779_103152 (*sdmA*) expression plasmid<br><br>Ga0097779_103152 was amplified by PCR with primers AL99 and AL100. The fragment was assembled by SLIC into the NcoI site of pABB466. Sequence was confirmed with oligos AL22 and AL23.	(*)
pBAD1031K_ESA_*sdmB* (pAL7169)	Ga0097779_103151 (*sdmB*) expression plasmid	

	Ga0097779_103151 was amplified by PCR with primers AL95 and AL96. The fragment was assembled by SLIC into the NcoI site of pABB466. Sequence was confirmed with oligos AL22 and AL23.	(*)
pBAD1031K_ESA_*sdmC* (pAL7180)	Ga0097779_109097 (*sdmC*) expression plasmid<br><br>Ga0097779_103152 was amplified by PCR with primers AL142 and AL143. The fragment was assembled by SLIC into the XbaI site of pABB466. Sequence was confirmed with oligos AL22 and AL23.	(*)
pBAD1031K_ESA_*sdmAB* (pAL7141)	Ga0097779_103152 (*sdmA*) and Ga0097779_103151 (*sdmB*) co-expression plasmid<br><br>Ga0097779_103152 and Ga0097779_103151 were amplified by PCR with primers AL99, AL111 and AL96 and AL112, respectively. Fragments were annealed using overlap extension PCR and assembled by SLIC into the NcoI site of pABB466. Sequence was confirmed with oligos AL22, AL23, AL34 and AL35.	(*)
pBAD1031K_ESA_*sdmAC* (pAL7181)	Ga0097779_103152 (sdmA) and Ga0097779_109097(sdmC) co-expression plasmid<br><br>Ga0097779_109097 was amplified using oligos AL142 and AL143. The fragment was assembled by SLIC into the XbaI site of pAL7134. Sequence was confirmed with oligos AL22 and AL161.	(*)
pBAD1031K_ESA_*sdmAB-sdmC* (pAL7170)	Ga0097779_103152 (sdmA), Ga0097779_103151 (sdmB), and Ga0097779_109097(sdmC) co-expression plasmid<br><br>Ga0097779_109097 was amplified using oligos AL142 and AL143. The fragment was assembled by SLIC into the XbaI site of pAL7141. Sequence was confirmed with oligos AL22, AL23 and AL161.	(*)
pBAD1031K_CALO_*sdmA* (pAL7298)	Ga0263810_115380 (sdmA) expression plasmid	

	Ga0263810_115380 was amplified by PCR with primers AL198 and AL258. The fragment was assembled by SLIC into the NcoI site of pABB466. Sequence was confirmed with oligos AL22 and AL23.	(*)
pBAD1031K_CALO_*sdmB* (pAL7296)	Ga0263810_115382 (*sdmB*) expression plasmid<br><br>Ga0263810_115382 was amplified by PCR with primers AL199 and AL260. The fragment was assembled by SLIC into the NcoI site of pABB466. Sequence was confirmed with oligos AL22 and AL23.	(*)
pBAD1031K_CALO_*sdmAB* (pAL7297)	Ga0263810_115380 (*sdmA*) and Ga0263810_115382 (*sdmB*) co-expression plasmid<br><br>Ga0263810_115380 and Ga0263810_115382 were amplified by PCR with primers AL198, AL259 and AL199 and AL261, respectively. Fragments were annealed using overlap extension PCR and assembled by SLIC into the NcoI site of pABB466. Sequence was confirmed with oligos AL22, AL23 and AL200.	(*)
pSRK_CALO_*osc* (pAL7309)	Ga0263810_115390 (*osc*) expression plasmid<br><br>Ga0263810_115390 was amplified by PCR with primers AL264 and AL265. The fragment was assembled by SLIC into the NdeI site of pABB492. Sequence was confirmed with oligos AL5 and AL7.	(*)

Supplementary Table 7. Heterologous expression strains used in this study. All expression strains are *E. coli* DH10B with pJBEI2997 (pABB302, CmR), pTrc (pABB278 or derivatives, AmpR), pSRK (pABB492 or derivatives, Gmr) (where indicated), and pBAD1031K (pABB466 or derivatives, KanR) (where indicated).

Expression Strain	Plasmids
PVW 7011	pJBEI2997 (pABB302), pTrc-*sqs*-synRBS-*osc*-synRBS-*smo* (pABB501), pSRKGm-lacUV5-rbs5 (pABB492), pBAD1031K (pABB466)
PVW 7130	pJBEI2997 (pABB302), pTrc-*sqs*-synRBS-*osc*-synRBS-*smo* (pABB501), pBAD1031K_ESA_*sdmA* (pAL7134), pSRK_MCAT_*sdmB* (pAL7003)
PVW 7131	pJBEI2997 (pABB302), pTrc-*sqs*-synRBS-*osc*-synRBS-*smo* (pABB501), +pBAD ESA 1017 + pSRK_MCAT_*sdmA* (pAL7004)
PVW 7305	pJBEI2997 (pABB302), pTrc-*sqs*-synRBS-*osc*-synRBS-*smo* (pABB501), pSRKGm-lacUV5-rbs5 (pABB492), pBAD1031K_ESA_*sdmA* (pAL7134)
PVW 7119	pJBEI2997 (pABB302), pTrc-*sqs*-synRBS-*osc*-synRBS-*smo* (pABB501), pSRKGm-lacUV5-rbs5 (pABB492), pBAD1031K_ESA_*sdmB* (pAL7169)
PVW 7228	pJBEI2997 (pABB302), pTrc-*sqs*-synRBS-*osc*-synRBS-*smo* (pABB501), pSRKGm-lacUV5-rbs5 (pABB492), pBAD1031K_ESA_*sdmC* (pAL7180)
PVW 7143	pJBEI2997 (pABB302), pTrc-*sqs*-synRBS-*osc*-synRBS-*smo* (pABB501), pSRKGm-lacUV5-rbs5 (pABB492), pBAD1031K_ESA_*sdmAB* (pAL7141)
PVW 7303	pJBEI2997 (pABB302), pTrc-*sqs*-synRBS-*osc*-synRBS-*smo* (pABB501), pSRKGm-lacUV5-rbs5 (pABB492), pBAD1031K_ESA_*sdmAC* (pAL7181)
PVW 7304	pJBEI2997 (pABB302), pTrc-*sqs*-synRBS-*osc*-synRBS-*smo* (pABB501), pSRKGm-lacUV5-rbs5 (pABB492), pBAD1031K_ESA_*sdmAB-sdmC* (pAL7170)
PVW 7301	pJBEI2997 (pABB302), pTrc-*sqs*-synRBS-*osc*-synRBS-*smo* (pABB501), pSRKGm-lacUV5-rbs5 (pABB492), pBAD1031K_CALO_*sdmA* (pAL7298)
PVW 7300	pJBEI2997 (pABB302), pTrc-*sqs*-synRBS-*osc*-synRBS-*smo* (pABB501), pSRKGm-lacUV5-rbs5 (pABB492), pBAD1031K_CALO_*sdmB* (pAL7296)
PVW 7299	pJBEI2997 (pABB302), pTrc-*sqs*-synRBS-*osc*-synRBS-*smo* (pABB501), pSRKGm-lacUV5-rbs5 (pABB492), pBAD1031K_CALO_*sdmAB* (pAL7297)
PVW 7310	pJBEI2997 (pABB302), pTrc-*sqs*-synRBS-*osc*-synRBS-*smo* (pABB501), pSRK_CALO_*osc* (pAL7309), pBAD1031K (ABB466)

Supplementary References:

1. Mitsche, M. A., McDonald, J. G., Hobbs, H. H. & Cohen, J. C. Flux analysis of cholesterol biosynthesis in vivo reveals multiple tissue and cell-type specific pathways. *eLife* **4**, e07999 (2015).
2. Lee, A. K. et al. C-4 sterol demethylation enzymes distinguish bacterial and eukaryotic sterol synthesis. Proceedings of the National Academy of Sciences **115**, 5884–5889 (2018).
3. Banta, A. B., Wei, J. H., Gill, C. C. C., Giner, J.-L. & Welander, P. V. Synthesis of arborane triterpenols by a bacterial oxidosqualene cyclase. Proc Natl Acad Sci U S A **114**, 245–250 (2017).
4. Chakravartty, V. & Cronan, J. E. A series of medium and high copy number arabinose-inducible Escherichia coli expression vectors compatible with pBR322 and pACYC184. *Plasmid* **81**, 21–26 (2015).
