# Supplementary material for: De novo cholesterol biosynthesis in bacteria
Source: Nat Commun. 2023 May 22;14:2904. doi: 10.1038/s41467-023-38638-8 (PMC10202945; doi:10.1038/s41467-023-38638-8)
Supplement: Supplementary file 3 — Description of Additional Supplementary Files [file 41467_2023_38638_MOESM3_ESM.pdf]

Description of Additional Supplementary Files:

Supplementary Data 1: Eukaryotic sterol biosynthesis pathway homologs in bacteria. We conducted a BLASTp search of bacteria in the genomic databases on the JGI IMG portal for oxidosqualene cyclase (1 x e -50, 30%ID). Of this subset of bacteria, we conducted further BLASTp searches using the putative cholesterol biosynthesis genes from E. salina and Calothrix. Cultured bacteria are bolded. IMG locus tags are listed for and highlighted green for organisms which harbor homologs. SMO, squalene monooxygenase; OSC, oxidosqualene cyclase; CYP51, C-14 demethylase; SdmA, sterol demethylase A; SdmB, Sterol demethylase B; SdmC, sterol demethylase C.
