# Supplementary material for: De novo cholesterol biosynthesis in bacteria
Source: Nat Commun. 2023 May 22;14:2904. doi: 10.1038/s41467-023-38638-8 (PMC10202945; doi:10.1038/s41467-023-38638-8)
Supplement: Supplementary file 5 — Reporting Summary [file 41467_2023_38638_MOESM5_ESM.pdf]

Corresponding author(s): Paula V. Welander

Last updated by author(s): May04,2023

Reporting Summary

Nature Portfolio wishes to improve the reproducibility of the work that we publish. This form provides structure for consistency and transparency in reporting. For further information on Nature Portfolio policies, see our Editorial Policies and the Editorial Policy Checklist.

Statistics

For all statistical analyses, confirm that the following items are present in the figure legend, table legend, main text, or Methods section.

n/a	Confirmed	
☒	☐	The exact sample size (*n*) for each experimental group/condition, given as a discrete number and unit of measurement
☒	☐	A statement on whether measurements were taken from distinct samples or whether the same sample was measured repeatedly
☒	☐	The statistical test(s) used AND whether they are one- or two-sided<br>*Only common tests should be described solely by name; describe more complex techniques in the Methods section.*
☒	☐	A description of all covariates tested
☒	☐	A description of any assumptions or corrections, such as tests of normality and adjustment for multiple comparisons
☒	☐	A full description of the statistical parameters including central tendency (e.g. means) or other basic estimates (e.g. regression coefficient) AND variation (e.g. standard deviation) or associated estimates of uncertainty (e.g. confidence intervals)
☒	☐	For null hypothesis testing, the test statistic (e.g. *F*, *t*, *r*) with confidence intervals, effect sizes, degrees of freedom and *P* value noted<br>*Give P values as exact values whenever suitable.*
☒	☐	For Bayesian analysis, information on the choice of priors and Markov chain Monte Carlo settings
☒	☐	For hierarchical and complex designs, identification of the appropriate level for tests and full reporting of outcomes
☒	☐	Estimates of effect sizes (e.g. Cohen's *d*, Pearson's *r*), indicating how they were calculated

Our web collection on statistics for biologists contains articles on many of the points above.

Software and code

Policy information about availability of computer code

Data collection	No code was used to collect data for this study.
Data analysis	Agilent MassHunter Qualitative Analysis B.06.00, breseq v0.35.4, MEGA 11.0.10, Bclconvert (v.3.9.3)

For manuscripts utilizing custom algorithms or software that are central to the research but not yet described in published literature, software must be made available to editors and reviewers. We strongly encourage code deposition in a community repository (e.g. GitHub). See the Nature Portfolio guidelines for submitting code & software for further information.

Data

Policy information about availability of data

All manuscripts must include a data availability statement. This statement should provide the following information, where applicable:

- Accession codes, unique identifiers, or web links for publicly available datasets
- A description of any restrictions on data availability
- For clinical datasets or third party data, please ensure that the statement adheres to our policy

Data that supports the findings of this study are available in the main text and supplement. Raw spectra and chromatogram data are provided in a separate source data file. Gene IDs are provided in supplementary table 2.. JGI IMG database (https://img.jgi.doe.gov/) is referenced in the data availability statement and accessible link included.

Human research participants

Policy information about studies involving human research participants and Sex and Gender in Research.

Reporting on sex and gender	There were no human research participants in this study
Population characteristics	There were no human research participants in this study
Recruitment	There were no human research participants in this study
Ethics oversight	There were no human research participants in this study

Note that full information on the approval of the study protocol must also be provided in the manuscript.

Field-specific reporting

Please select the one below that is the best fit for your research. If you are not sure, read the appropriate sections before making your selection.

☒ Life sciences ☐ Behavioural & social sciences ☐ Ecological, evolutionary & environmental sciences

For a reference copy of the document with all sections, see nature.com/documents/nr-reporting-summary-flat.pdf

Life sciences study design

All studies must disclose on these points even when the disclosure is negative.

Sample size	No sample size calculations were necessary. The goal of this study is to demonstrate the presence or absence of sterol intermediates under specified growth conditions. Replicates were sufficient to determine presence or absence and no statistical analyses were necessary.
Data exclusions	No data was excluded from the manuscript.
Replication	For E. salina all sterol extractions were performed in triplicate and reproducible. Upon serial passaging Calothrix, the strain stopped sterol production. This strain is no longer commercially available and we were unable to reproduce our initial analyses. Genome sequencing and heterolgous expression showed that cholesterol biosynthesis genes are still present and functional when expressed. This is discussed in the main text, supplement and reviews of the paper.
Randomization	There are no experimental groups in our study.
Blinding	There is no group allocation in our study.

Reporting for specific materials, systems and methods

We require information from authors about some types of materials, experimental systems and methods used in many studies. Here, indicate whether each material, system or method listed is relevant to your study. If you are not sure if a list item applies to your research, read the appropriate section before selecting a response.

Materials & experimental systems

n/a	Involved in the study
☒	☐ Antibodies
☒	☐ Eukaryotic cell lines
☒	☐ Palaeontology and archaeology
☒	☐ Animals and other organisms
☒	☐ Clinical data
☒	☐ Dual use research of concern

Methods

n/a	Involved in the study
☒	☐ ChIP-seq
☒	☐ Flow cytometry
☒	☐ MRI-based neuroimaging
